# A Series of An^VI^O_2_^2+^ Complexes (An = U, Np, Pu) with N_3_O_2_-Donating Schiff-Base Ligands: Systematic Trends in the Molecular Structures and Redox Behavior

**DOI:** 10.1021/acs.inorgchem.4c04185

**Published:** 2025-01-03

**Authors:** Tomoyuki Takeyama, Satoru Tsushima, Robert Gericke, Tamara M. Duckworth, Peter Kaden, Juliane März, Koichiro Takao

**Affiliations:** † Department of Applied Chemistry, Sanyo-Onoda City University, 1-1-1, Daigakudori, Sanyo-Onoda, Yamaguchi 756-0884, Japan; ‡ Laboratory for Zero-Carbon Energy, Institute of Integrated Research, Institute of Science Tokyo, 2-12-1 N1-32, O-okayama, Meguro-ku, Tokyo 152-8550, Japan; § Institute of Resource Ecology, Helmholtz-Zentrum Dresden-Rossendorf (HZDR) Bautzner Landstraße 400, Dresden 01328, Germany

## Abstract

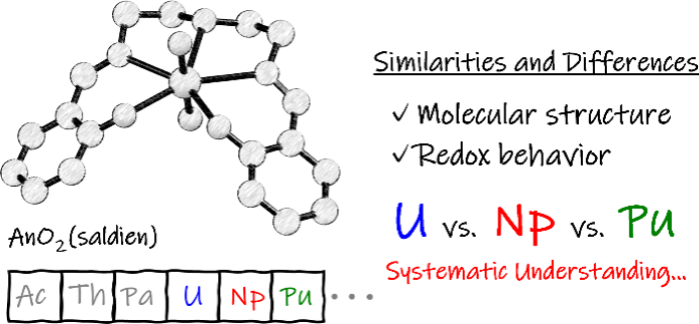

In their + V and + VI oxidation states, actinide elements (U, Np, and Pu) are commonly encountered in characteristic linear dioxo structures, known as actinyl ions (AnO_2_*^n^*^+^; An = U, Np, Pu, *n* = 1, 2). A systematic understanding of the structural and redox behavior of An^V^O_2_^+^/An^VI^O_2_^2+^ complexes is expected to provide valuable information for controlling the behavior of An elements in natural environments and in nuclear fuel cycles while enabling the development of spintronics and new reactivities that utilize the anisotropic spin of the 5f electrons. However, systematic trends in the behavior of An^V^O_2_^+^/An^VI^O_2_^2+^ complexes remain poorly understood. The [An^V/VI^O_2_(saldien)]^−/0^ complexes (saldien^2–^ = *N,N’*-disalicylidenediethylenetriamine) studied here offer a promising avenue for advancing our understanding of this subject. The molecular structures of a series of [An^VI^O_2_(saldien)] complexes were found to exhibit notable similarities through these An elements with minor, but still significant, contributions from the actinide contraction. The redox potentials of the [An^V/VI^O_2_(saldien)]^−/0^ couples clearly increase from U to Np, followed by a subsequent decrease from Np to Pu (−1.667 V vs Fc^0/+^ for [U^V/VI^O_2_(saldien)]^−/0^, −0.650 V for [Np^V/VI^O_2_(saldien)]^−/0^ and −0.698 V for [Pu^V/VI^O_2_(saldien)]^−/0^). Such a difference can be explained in terms of the difference in character of the electronic configuration of the + VI oxidation state. A series of these redox trends was also successfully reproduced by DFT-based calculations. These findings provide valuable information for controlling the oxidation states of the An elements.

## Abstract 2

A series of [An^VI^O_2_(saldien)] complexes (An = U, Np, Pu) were synthesized and characterized. The molecular structures of these [An^VI^O_2_(saldien)] complexes in the crystalline state are comparable. In contrast, the redox potentials between the [An^V/VI^O_2_(saldien)]^−/0^ couples strongly depend on the nature of the central actinide elements. The redox potentials of the [Np^V/VI^O_2_(saldien)]^−/0^ and [Pu^V/VI^O_2_(saldien)]^−/0^ couples are comparable, while the potential of the [U^V/VI^O_2_(saldien)]^−/0^ couple is significantly more negative than that of the other [An^V/VI^O_2_(saldien)]^−/0^ couples.

## Introduction

A more profound understanding of the complexation behavior and redox properties of the actinide elements is crucial for comprehending actinide migration in natural environments and for formulation of strategies for nuclear fuel recycling.^1^ The + V and + VI oxidation states of the actinide elements such as U, Np, and Pu are frequently found in nuclear engineering either as stable and predominant species or as reaction intermediates through redox chemistry. The An^V^ and An^VI^ states (An = U, Np, Pu) typically exist as linear dioxo structures known as actinyl ions (AnO_2_*^n^*^+^; An = U, Np, Pu).^2^ Therefore, elucidating the systematic trends in the coordination chemistry and redox chemistry of the An^V^O_2_^+^/An^VI^O_2_^2+^ couple will provide a better understanding of the behavior of actinide elements in the environment and in the nuclear fuel cycle. However, systematic studies^3,4^ on the coordination and redox chemistry of An^V^O_2_^+^/An^VI^O_2_^2+^ species remain scarce to date, likely due to the inherent difficulties associated with their radioactivity, particularly for Np and Pu.

Uranyl complexes are the most well-studied An^V^O_2_^+^/An^VI^O_2_^2+^ complexes as they are relatively easy to handle compared to other An^V^O_2_^+^/An^VI^O_2_^2+^ complexes. The coordination and redox chemistry of U^V^O_2_^+^/U^VI^O_2_^2+^ complexes have been extensively investigated.^5−22^ Over the past two decades, the most prominent area of research in this field has been the stabilization of U^V^O_2_^+^ complexes with a 5f^1^ electronic configuration.^7−9,18−22^ The presence of unpaired 5f electron gives rise to a range of functions and reactivities that cannot be achieved by transition-metal complexes.^6,15,16,23−27^ However, U^V^O_2_^+^ species are typically unstable due to autoxidation upon contact with O_2_, and also suffer from disproportionation to afford U^VI^O_2_^2+^ and U^4+^.^28−30^ In the latter reaction, the oxygen atoms in U^V^O_2_^+^ tend to interact with the U atom of another U^V^O_2_^+^ species, a phenomenon known as cation–cation interaction (CCI). CCI facilitates inner-sphere electron transfer from one U^5+^ center to the other, ultimately yielding U^VI^O_2_^2+^ and U^4+^.^28−30^ Thus, CCI contributes to the destabilization of U^V^O_2_^+^. To stabilize such U^V^O_2_^+^ species, it is essential to prevent CCI between U^V^O_2_^+^ ions. The use of Schiff-base ligands such as *N,N’*-disalicylidene-*o*-phenylenediamine (salophen^2–^)^18^ and *N,N’*-disalicylidenediethylenetriamine (saldien^2–^)^19^ represent early examples of the successful stabilization of U^V^O_2_^+^ species that prevent CCI by strong coordination through the chelate effect. Today, the use of Schiff-base ligands in the formation of U^V^O_2_^+^/U^VI^O_2_^2+^ complexes has been instrumental in the development of uranium-based single-molecule magnets^24−27^ as well as in the recovery of uranium from seawater.^31^

**Figure 1:**
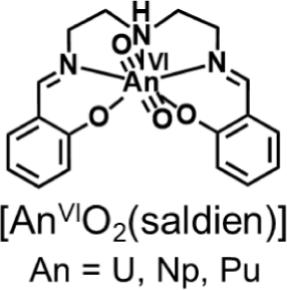
Structure of the [An^VI^O_2_(saldien)] complexes (An = U, Np, Pu) examined in this study.

The chemical properties of An^V^O_2_^+^/An^VI^O_2_^2+^ complexes (An = U, Np, Pu) are typically considered to have significant similarities. However, some recent examples have shown that the redox behavior of U^V^O_2_^+^/U^VI^O_2_^2+^ and Np^V^O_2_^+^/Np^VI^O_2_^2+^ complexes with Schiff-base ligands strongly depends on the central actinide element.^32^ In systems that utilize *t*Bu-pdiop^2–^ (2,6-bis[*N*-(3,5-di-*tert*-butyl-2-hydroxyphenyl)iminomethyl]pyridine) ligands, the U^VI^O_2_^2+^ complex exhibits remarkable stability,^23^ whereas the Np^VI^O_2_^2+^ complex undergoes rapid conversion to the corresponding Np^IV^ complex.^32^ Identifying similarities and differences in the chemistry of An^V^O_2_^+^/An^VI^O_2_^2+^ complexes represents a crucial initial step toward attaining a comprehensive understanding of these complexes. In this study, we aimed to identify and rationalize systematic trends in the structures and redox behavior of a series of An^V^O_2_^+^/An^VI^O_2_^2+^ complexes. In order to gain an accurate understanding of the redox behavior of An^V^O_2_^+^/An^VI^O_2_^2+^ complexes, it will be necessary to prevent CCIs in An^V^O_2_^+^ and establish redox systems between mononuclear An^VI^O_2_^2+^ and An^V^O_2_^+^ complexes. However, some reported Schiff-base Np^V^O_2_^+^ complexes are oligomeric or polymeric due to CCIs between Np^V^O_2_^+^ centers.^33,34^ A pentadentate N_3_O_2_-donating Schiff-base ligand, saldien^2–^, which we have previously reported,^20^ could potentially be effective in preventing CCIs and thus stabilize mononuclear An^V^O_2_^+^ complexes as shown in Figure 1. It thus appears that the use of the saldien^2–^ ligand may facilitate a systematic understanding of the molecular structures and redox properties of a series of An^V^O_2_^+^/An^VI^O_2_^2+^ complexes (An = U, Np, Pu).

In this study, we synthesized and characterized [Np^VI^O_2_(saldien)] and [Pu^VI^O_2_(saldien)] (Figure 1) and compared their molecular structures to that of the U^VI^O_2_^2+^ analogue, which we have previously reported.^20^ Furthermore, the redox behavior of a series of [An^VI^O_2_(saldien)] complexes (An = U, Np, Pu) were examined based on a combination of experimental and computational methods. We also discuss the trends observable in the redox behavior of the [An^VI^O_2_(saldien)] complexes.

## Experimental Section

*Caution!*^237^Np and ^242^Pu are highly radioactive α-emitters with half-live times of 2.14 × 10^6^ and 3.76 × 10^5^ years, respectively. ^237^Np and ^242^Pu must be handled in dedicated facilities with appropriate equipment for radioactive materials in order to avoid health risks caused by radiation exposure. All operations to handle Np and Pu were conducted in a dedicated glovebox in a controlled laboratory at the Institute of Resource Ecology, Helmholtz-Zentrum Dresden-Rossendorf.

### Materials and Syntheses

All preparations were performed under a rigorously controlled atmosphere free of moisture and oxygen in a nitrogen-filled glovebox. The solvents used were dried using the solvent purification system MBraun SPS 5 and stored over molecular sieves (3 Å) prior to use. Unless specified otherwise, all reagents were of reagent grade and used as received. H_2_(saldien) was synthesized as reported elsewhere.^20^ Neptunyl(VI) nitrate hydrate (Np^VI^O_2_(NO_3_)_2_·*n*H_2_O, *n* ∼ 5) was prepared by dissolving NpO_2_ (lab-made) in HNO_3_ (aq.). The solution was heated to reflux for 48 h, and concentrated to near dryness. The obtained Np^VI^O_2_(NO_3_)_2_·*n*H_2_O residue was then added to a small quantity of water and concentrated to near dryness. Plutonyl(VI) nitrate hydrate (Pu^VI^O_2_(NO_3_)_2_·*n*H_2_O, *n* ∼ 5) was prepared by dissolving PuO_2_ (lab-made) in HNO_3_ (aq.). This solution was heated to reflux for 48 h, and concentrated to near dryness. The obtained Pu^VI^O_2_(NO_3_)_2_·*n*H_2_O residue was added to a small amount of water, and concentrated to near dryness.

#### [Np^VI^O_2_(saldien)]

An ethanol solution of H_2_(saldien) (0.1 M, 100 μL) was added to an ethanol solution of Np^VI^O_2_(NO_3_)_2_·*n*H_2_O (0.1 M, 100 μL) and a gray precipitate was formed immediately. The supernatant was removed, and the precipitate was washed with ethanol. Recrystallization from pyridine/pentane yielded black crystalline blocks; yield: 3.0 mg (52%).

#### [Pu^VI^O_2_(saldien)]

An ethanol solution of H_2_(saldien) (0.1 M, 200 μL) was added to an ethanol solution of Pu^VI^O_2_(NO_3_)_2_·*n*H_2_O (0.1 M, 200 μL) and a black-green precipitate formed immediately. The supernatant was removed, and the precipitate was washed with ethanol. Recrystallization from pyridine/diethyl ether yielded green crystalline plates; yield: 7.6 mg (65%).

### Methods

IR measurements were performed on an Agilent Cary 630 FTIR spectrometer equipped with a single-reflection attenuated total reflection (ATR) accessory made of diamond. The ^1^H NMR and ^1^H–^1^H COSY spectra were recorded with a Varian Inova 400 spectrometer (^1^H: 399.89 MHz). Each sample solution was loaded in an NMR tube equipped with the J.Young-valve. The UV-vis-NIR absorption spectra of [Np^VI^O_2_(saldien)] and [Pu^VI^O_2_(saldien)] in pyridine were collected at 295 K using an Avantes AvaSpec-ULS2048 StarLine Versatile Fiber-optic spectrometer connected with optical fibers to a cuvette housing in the glovebox. Cyclic voltammetry measurements of [Np^VI^O_2_(saldien)] (1 mM) and [Pu^VI^O_2_(saldien)] (0.8 mM) dissolved in pyridine containing 0.1 M tetra-*n*-butylammonium perchlorate (TBAP) were performed at 295 K under a dry nitrogen atmosphere using a CHI 650C electrochemical analyzer. A three-electrode system consisting of a Pt-disk working electrode, a Pt-wire counter electrode, and an Ag^0/+^ reference electrode (0.1 M TBAP + 1 mM AgNO_3_/CH_3_CN) was used. The ferrocene/ferrocenium ion redox couple (Fc^0/+^) was taken as the external standard redox system. All samples were prepared under an inert nitrogen atmosphere.

### Crystallographic Analysis

X-ray diffraction data of well-shaped single crystals of [Np^VI^O_2_(saldien)] and [Pu^VI^O_2_(saldien)] were collected on a Bruker D8 VENTURE diffractometer equipped with a Photon II 7 array detector using mirror optics monochromator Mo *Kα* radiation (λ = 0.71073 Å). Each sample was mounted on a MiTeGen Dual Thickness MicroMount and placed in a temperature-controlled N_2_ gas flow. The computer programs SMART and SAINT, which are implemented in the APEX4 software suite, were used for data collection in φ- and ω-scan modes and data processing, respectively. Absorption correction was applied by using the strong-absorber option of SADAS. Structures were solved by direct methods and refined anisotropically using the SHELX program suite^35^ for non-hydrogen atoms via full-matrix least-squares calculations. Each refinement was continued until all shifts were smaller than one-third of the standard deviations of the parameters involved. Hydrogen atoms were located at the calculated positions. All hydrogen atoms were constrained to an ideal geometry with C–H = 0.95 Å. The thermal parameters of all hydrogen atoms were related to those of their parent atoms by the equation *U*(H) = 1.2*U*eq(C,N). All calculations were performed using the *Olex2* crystallographic software program package.^36^

Crystallographic data for [Np^VI^O_2_(saldien)]: *F*w = 578.36, 0.19 × 0.13 × 0.07 mm^3^, orthorhombic, *Pnma*, *a* = 10.4494(4) Å, *b* = 21.3176(8) Å, *c* = 7.9784(3) Å, α = β = γ = 90.0°, *V* = 1777.24(12) Å^3^, *Z* = 4, *T* = 100 K, *D*_calcd_ = 2.162 g/cm^3^, μ(Mo *Kα*) = 5.876 mm^–1^, *GOF* = 1.143, *R*_1_(*I* > 2σ) = 0.0125, *wR*_2_(all) = 0.0286.

Crystallographic data for [Pu^VI^O_2_(saldien)]: *F*w = 583.36, 0.12 × 0.12 × 0.03 mm^3^, orthorhombic, *Pnma*, *a* = 10.5281(7) Å, *b* = 21.5408(14) Å, *c* = 7.8214(5) Å, α = β = γ = 90.0°, *V* = 1773.8(2) Å^3^, *Z* = 4, *T* = 100 K, *D*_calcd_ = 2.184 g/cm^3^, μ(Mo *Kα*) = 3.745 mm^–1^, *GOF* = 1.193, *R*_1_(*I* > 2σ) = 0.0257, *wR*_2_(all) = 0.0543.

### Theoretical Calculations

DFT calculations were performed using the Gaussian 16 program (Revision B.01) suite.^37^ The atomic coordinates of the [An^VI^O_2_(saldien)] complexes were taken from those determined by single-crystal X-ray diffraction analysis. The structures of [U^VI^O_2_(saldien)], [Np^VI^O_2_(saldien)], and [Pu^VI^O_2_(saldien)] were optimized in singlet, doublet and triplet states, respectively. In a similar manner, the structure optimization of each reduced complex, [AnO_2_(saldien)]^−^, was performed following an addition of a single electron to the structure obtained for the corresponding [An^VI^O_2_(saldien)] complex and assuming doublet, triplet, and quintet spin state for [UO_2_(saldien)]^−^, [NpO_2_(saldien)]^−^, and [PuO_2_(saldien)]^−^, respectively. The B3LYP^38^ hybrid functional was employed. For uranium, neptunium and plutonium, the effective core potential as well as corresponding basis sets developed by Dolg et al. (Stuttgart/Cologne RSC ECP) were employed.^39^ The most diffuse basis functions on actinide with the exponent 0.005 (all s-, *p*-, d-, and f-type functions) were omitted as in previous studies.^40,41^ The 6-311G(d) basis sets were used for other elements (C, H, N, O). Solvation effects were included in the calculations by using a conductor-like polarized continuum model (CPCM) (dielectric constant: 12.978).^42,43^ Vibrational frequency calculations at the same level of theory confirmed the absence of any imaginary frequencies. Single-point calculations for an energetic analysis were performed on the optimized geometries using the same conditions. The Mulliken spin-density plots and MOs were visualized using GaussView 6.1.^44^ Time-dependent DFT (TD-DFT) calculations were performed using the ORCA program (version 4.0.1)^45^ based on the DFT-optimized structures obtained from the Gaussian 16 calculations. The all-electron SARC-ZORA-TZVP, segmented all-electron relativistically contracted (SARC) basis sets with a zero-order regular approximation (ZORA) Hamiltonian that considers the scalar-relativistic term was used for the actinides. For the remaining elements, the Def2-TZVP (triple-ζ valence basis sets with a polarized function) basis set was applied. Theoretical electronic-transition spectra were recorded for low-lying excited states (>300 nm).

## Results and Discussion

### Synthesis and Characterization of the [An^VI^O_2_(saldien)] Complexes

The reaction of actinyl(VI) nitrate hydrate (An^VI^O_2_(NO_3_)_2_·*n*H_2_O, *n* ∼ 5) with one equivalent of H_2_(saldien) in ethanol yielded [An^VI^O_2_(saldien)]. Following recrystallization, [Np^VI^O_2_(saldien)] was obtained as black crystalline blocks in 52% yield, while [Pu^VI^O_2_(saldien)] was obtained as green crystalline plates in 65% yield. The ^1^H NMR and ^1^H–^1^H COSY spectra (Figures S1–S3) indicated that [An^VI^O_2_(saldien)] were obtained as expected. The solid-state IR spectra of [An^VI^O_2_(saldien)] are shown in Figure S4. The [O≡Np^VI^≡O]^2+^ and [O≡Pu^VI^≡O]^2+^ asymmetric stretching vibrations (ν_3_) were observed at 887 and 892 cm^–1^, respectively. These values are in good agreement with the previously reported An^VI^O_2_^2+^ complexes.^4b^ The characteristic IR peaks are also observed for the previously reported [U^VI^O_2_(saldien)] complex (ν_3_ = 897 cm^–1^).^20^

**Figure 2:**
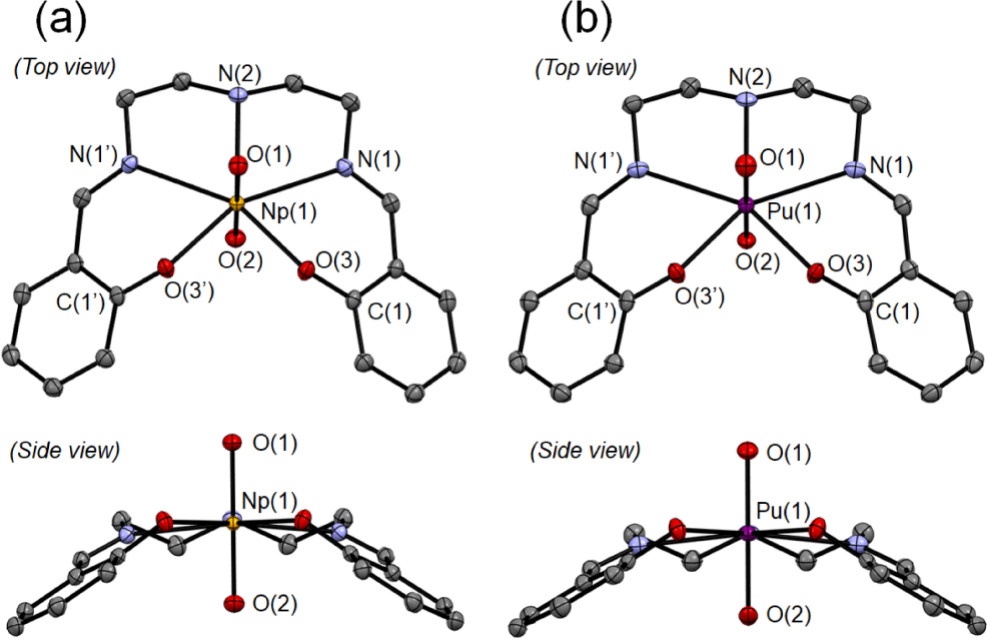
Molecular structures of (a) [Np^VI^O_2_(saldien)] and (b) [Pu^VI^O_2_(saldien)] in the crystalline state with thermal ellipsoids at 50% probability; all hydrogen atoms are omitted for clarity.

The molecular and crystal structures of the [An^VI^O_2_(saldien)] complexes were determined through X-ray crystallography. The resulting molecular structures of the [An^VI^O_2_(saldien)] complexes are shown in Figure 2. Selected bond lengths of the [An^VI^O_2_(saldien)] complexes are summarized in Table 1. In general, the molecular structures of both [An^VI^O_2_(saldien)] complexes show that the saldien^2–^ ligand is coordinated to the equatorial plane of the An^VI^O_2_^2+^ species, which results in a pentagonal bipyramidal coordination geometry. As anticipated, the saldien^2–^ ligand fully saturates the equatorial plane of the An^VI^O_2_^2+^ species, thereby precluding any further coordination, including CCIs, at the An center. Intermolecular hydrogen bonds are observed between neighboring complexes, i.e., N(2)–H(2)···O(2) and N(2)–H(2)···O(3), with distances of 2.63–2.71 Å. Such intermolecular hydrogen bonds were also observed in the crystal structure of [U^VI^O_2_(saldien)].^20^ As a matter of fact, the crystallographic parameters of [An^VI^O_2_(saldien)] (An = Np, Pu) explored here are nearly identical with those of [U^VI^O_2_(saldien)] (orthorhombic, *Pnma*, *a* = 10.464(3) Å, *b* = 21.617(6)Å, *c* = 7.976(2) Å). While [U^VI^O_2_(saldien)] may contain a DMSO molecule as a crystalline solvent to afford different crystal structures, no solvates of the Np and Pu analogues were confirmed probably due to use of different solvents for recrystallization (pyridine/pentane for Np, pyridine/diethyl ether for Pu).

**Table 1:** Selected Bond Lengths (Å) of [U^VI^O_2_(saldien)],^20^ [Np^VI^O_2_(saldien)], and [Pu^VI^O_2_(saldien)]

[U^VI^O_2_(saldien)]^20^		[Np^VI^O_2_(saldien)]		[Pu^VI^O_2_(saldien)]	
U(1)–O(1)	1.786(6)	Np(1)–O(1)	1.775(2)	Pu(1)–O(1)	1.767(4)
U(1)–O(2)	1.788(7)	Np(1)–O(2)	1.764(2)	Pu(1)–O(2)	1.761(4)
U(1)–O(3)	2.249(4)	Np(1)–O(3)	2.227(1)	Pu(1)–O(3)	2.246(3)
U(1)–N(1)	2.569(6)	Np(1)–N(1)	2.554(2)	Pu(1)–N(1)	2.538(3)
U(1)–N(2)	2.562(7)	Np(1)–N(2)	2.574(2)	Pu(1)–N(2)	2.533(5)
C(1)–O(3)	1.307(8)	C(1)–O(3)	1.318(2)	C(1)–O(3)	1.312(5)

The An^VI^≡O bond lengths of the [An^VI^O_2_(saldien)] complexes (An(1)–O(1) and An(1)–O(2)) are similar to the corresponding bond lengths of the An^VI^O_2_^2+^ complexes of Schiff-base ligands that have been reported previously (1.757–1.772 Å for Np^VI^≡O and 1.768–1.781 Å for Pu^VI^≡O).^34,46^ The average bond lengths between An(1)–O(1) and An(1)–O(2) were calculated to be 1.787 Å for [U^VI^O_2_(saldien)],^20^ 1.770 Å for [Np^VI^O_2_(saldien)], and 1.764 Å for [Pu^VI^O_2_(saldien)]. The decrease in An^V^≡O bond lengths observed in going from the U to the Pu species can be attributed to the actinide contraction, i.e., the decreasing ionic radius of the An atoms with increasing atomic number.^2^ A comparable reduction in bond length was observed for the An (1)–N(1) bond lengths going from U to Pu (Table 1). In contrast, the An(1)–O(3) and An(1)–N(2) bond lengths do not clearly exhibit a systematic trend corresponding to the actinide contraction. Most researchers in this field agree that covalency also contributes to the bonding interactions between early actinides and coordinated atoms.^47−49^ The An(1)–O(3) and An(1)–N(2) bond lengths may be affected by both the actinide contraction and the bond covalency.

### Electrochemistry and Spectroelectrochemistry of the [An^VI^O_2_(saldien)] Complexes

**Figure 3:**
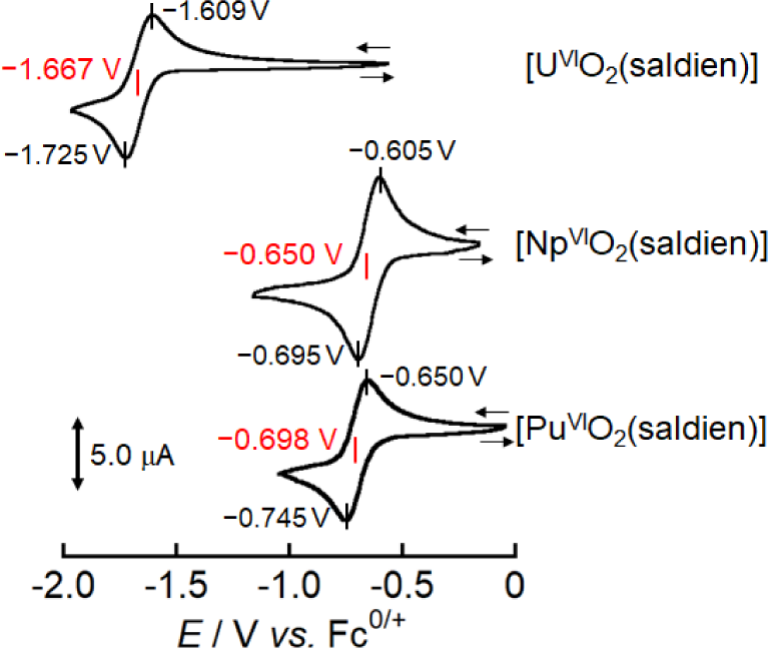
Cyclic voltammograms of [U^VI^O_2_(saldien)] (1 mM), [Np^VI^O_2_(saldien)] (1 mM), and [Pu^VI^O_2_(saldien)] (0.8 mM) in pyridine containing 0.1 M TBAP (*n*-tetrabutylammoniumperchlorate) at a sweep rate (*v*) of 100 mV·s^–1^.

We have previously reported that a [U^V/VI^O_2_(saldien)]^−/0^ redox process occurs at −1.582 V vs Fc^0/+^ in DMSO.^20^ In the present study, pyridine was employed as a solvent for electrochemistry to prepare 1 mM solutions of [Np^VI^O_2_(saldien)] and 0.8 mM solutions of [Pu^VI^O_2_(saldien)], as these complexes were poorly soluble in DMSO, and it was unable to obtain clear cyclic voltammograms therein. Prior to investigating the cyclic voltammograms of the [Np^VI^O_2_(saldien)] and [Pu^VI^O_2_(saldien)], we revisited that of [U^VI^O_2_(saldien)] (1 mM) in pyridine at 295 K. The cyclic voltammogram of [U^VI^O_2_(saldien)] (Figure 3) exhibited a set of cathodic (*E*_pc_) and anodic (*E*_pa_) peaks. The estimated formal potential (*E*°′ = (*E*_pc_ + *E*_pa_)/2) of the [U^V/VI^O_2_(saldien)]^−/0^ redox process was −1.667 V vs Fc^0/+^, which is by 85 mV more negative than the corresponding value in DMSO (−1.582 V).^20^

**Figure 4:**
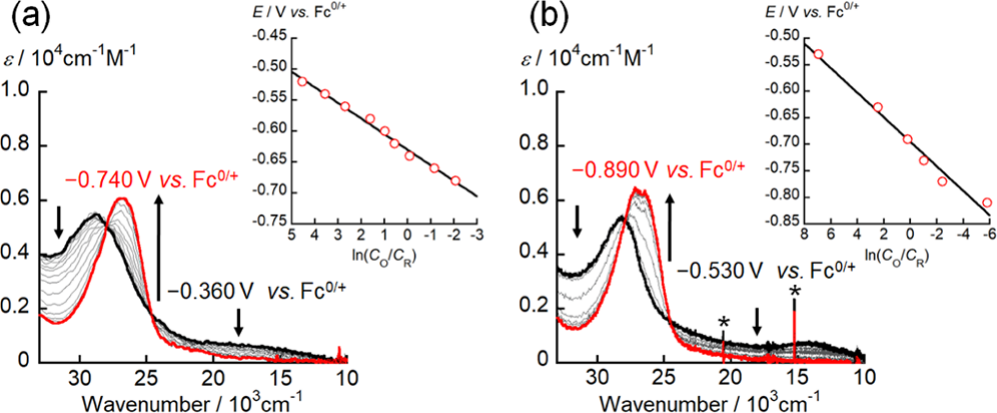
UV-vis-NIR spectral changes during the electrochemical reduction of (a) [Np^VI^O_2_(saldien)] and (b) [Pu^VI^O_2_(saldien)] recorded at different applied potentials at 295 K in pyridine containing 0.1 M TBAP. Black and red bold curves represent the absorption spectra of the [An^VI^O_2_(saldien)] and [An^V^O_2_(saldien)]^−^ complexes, respectively (An = Np and Pu). Inset: Nernstian plot calculated from the absorbance changes. Asterisk indicates noise resulting from the equipment.

The cyclic voltammograms of [Np^VI^O_2_(saldien)] and [Pu^VI^O_2_(saldien)] (Figure 3) also exhibit one *E*_pc_ and one *E*_pa_ peak, and the *E*°′ values were estimated to be −0.650 V vs Fc^0/+^ for [Np^VI^O_2_(saldien)] and −0.698 V vs Fc^0/+^ for [Pu^VI^O_2_(saldien)]. These redox potentials are significantly more positive than the *E*°′ value of the [U^V/VI^O_2_(saldien)]^−/0^ (−1.667 V) couple. The *E*°′ values demonstrates an increase from U to Np, followed by a subsequent decrease from Np to Pu. In order to determine the electron stoichiometry (*n*) of the redox processes observed for [Np^VI^O_2_(saldien)] and [Pu^VI^O_2_(saldien)], spectroelectrochemical measurements were conducted. For that purpose, the UV-vis-NIR spectra of [Np^VI^O_2_(saldien)] and [Pu^VI^O_2_(saldien)] in pyridine were recorded at a series of stepwise variation of applied potentials between −0.360 to −0.740 V vs Fc^0/+^ for [Np^VI^O_2_(saldien)] and −0.530 to −0.890 V vs Fc^0/+^ for [Pu^VI^O_2_(saldien)]. Figure 4 shows a series of UV-vis-NIR absorption spectra recorded at different potentials.

As a general trend, a decrease in the applied potentials was accompanied by a decrease in the intensities of the absorption bands at around 30000 and 24000–10000 cm^–1^, while new absorption bands appeared at 27000 and 10600 cm^–1^. The presence of several isosbestic points is clearly observed at around 28000 and 25000 cm^–1^, indicating that a redox equilibrium of [An^VI^O_2_(saldien)] (An = Np and Pu) solely occurs in the potential range studied here. The spectral change was observed to converge at −0.740 V vs Fc^0/+^ for [Np^VI^O_2_(saldien)] and −0.890 V vs Fc^0/+^ for [Pu^VI^O_2_(saldien)], implying that the reduction of [An^VI^O_2_(saldien)] was complete at this potential. The ratio (*C*_O_/*C*_R_) between the concentrations of the oxidant ([An^VI^O_2_(saldien)], *C*_O_) and the reductant (*C*_R_) at each potential was calculated using the absorbance change. The inset of Figure 4 shows the relationship between ln(*C*_O_/*C*_R_) and the applied potential (*E vs* Fc^0/+^), which should theoretically follow the Nernst eq (eq 1).
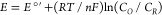
1where *R*, *T*, and *F* are the gas constant (8.314 J·mol^–1^·K^–1^), the absolute temperature (295 K), and the Faraday constant (96485 C·mol^–1^), respectively. The least-squares fit of eq 1 to the plot of *E* as a function of ln(*C*_O_/*C*_R_) allows *n* and *E*°′ for the observed redox reaction of [An^VI^O_2_(saldien)] to be determined. From the best-fit line in the inset of Figure 4, the obtained values are *n* = 1.0(3) and *E*°′ = −0.64(2) V vs Fc^0/+^ for [Np^VI^O_2_(saldien)] and *n* = 1.1(3) and *E*°′ = −0.70(2) V for [Pu^VI^O_2_(saldien)], respectively. Therefore, it seems feasible to conclude that the redox reactions of [An^VI^O_2_(saldien)] observed in the current systems of both An = Np, Pu are single electron processes, i.e., [An^VI^O_2_(saldien)] + e^–^ ⇌ [AnO_2_(saldien)]^−^. It should also be noted that the *E*°′ value estimated from the spectroelectrochemistry are consistent within an acceptable margin of error with the redox potentials determined through cyclic voltammetry (CV) measurements for each An system. In accordance with redox innocence of saldien^2–^ in the U analogue we reported previously,^20^ the reductant [AnO_2_(saldien)]^−^ observed here would be of pentavalent Np or Pu, although it is necessary to further explore redox activities of both An centers and ligands theoretically.

### Electronic Spectra of the [An^VI^O_2_(saldien)] and [AnO_2_(saldien)]^−^ Complexes

**Figure 5:**
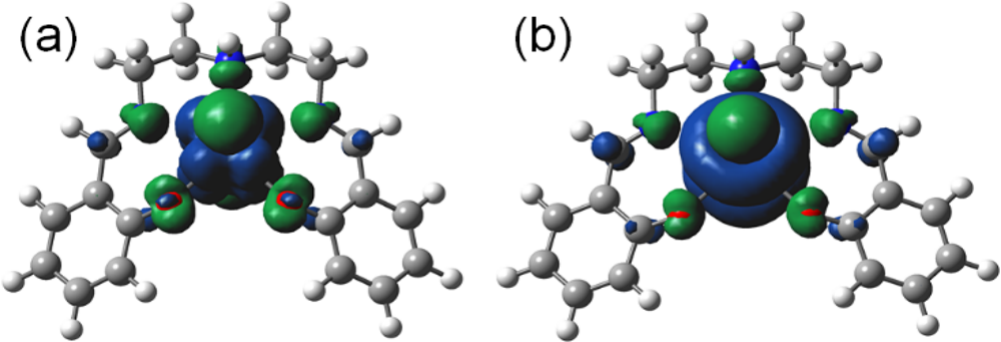
Spin-density plot of (a) [Np^VI^O_2_(saldien)] and (b) [Np^V^O_2_(saldien)]^−^.

**Figure 6:**
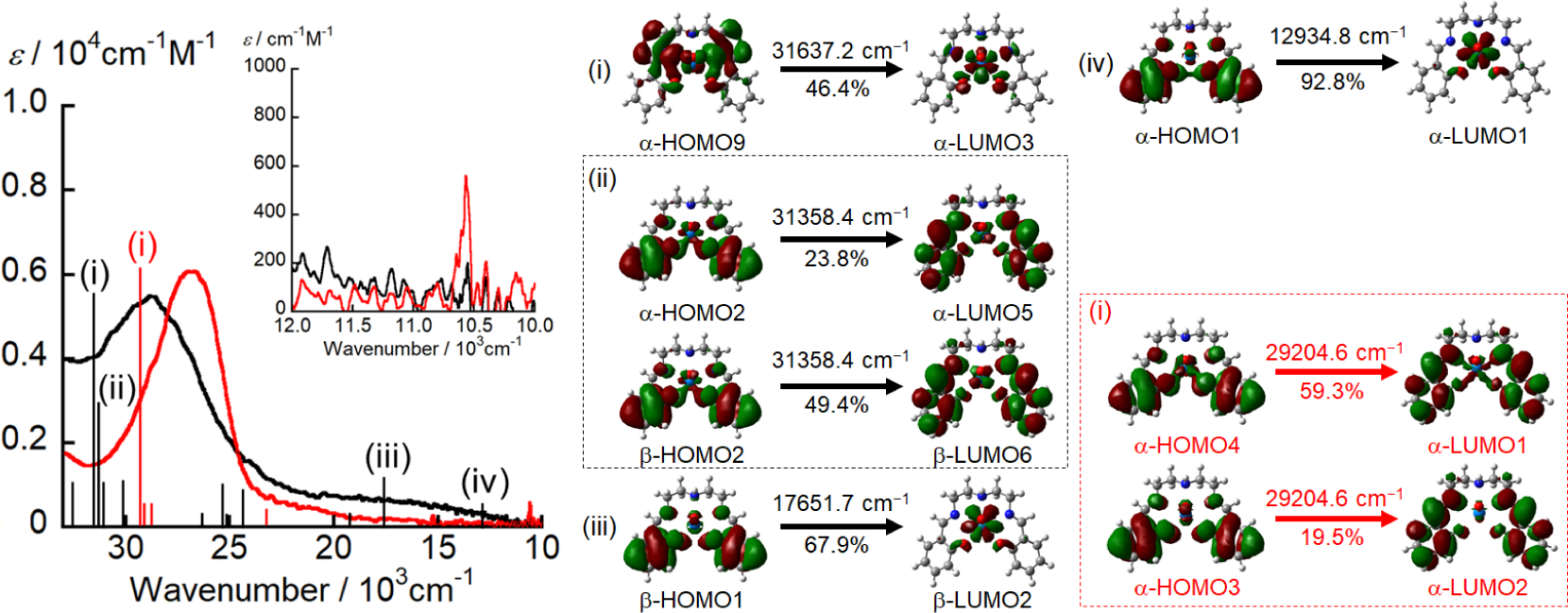
UV-vis-NIR spectra of [Np^VI^O_2_(saldien)] (black line) and [Np^V^O_2_(saldien)]^−^ (red line) in pyridine and the band positions and intensities predicted by the TD-DFT calculations. The vertical black and red lines correspond to the calculated transitions for [Np^VI^O_2_(saldien)] and [Np^V^O_2_(saldien)]^−^, respectively.

As shown in Figure 4a, the UV-vis-NIR spectrum of [Np^VI^O_2_(saldien)] shows an intense band at 29000 cm^–1^ (ε = 6000 M^–1^·cm^–1^) and a broad band in the 22000–11000 cm^–1^ spectral range (ε = 700 M^–1^·cm^–1^). These spectral features are analogous to those observed in other previously reported Np^VI^O_2_^2+^ complexes with Schiff-base ligands.^34,46^ To provide a better understanding of the UV-vis-NIR spectrum of [Np^VI^O_2_(saldien)] in pyridine, we optimized the structure of [Np^VI^O_2_(saldien)] using DFT calculations and studied its electronic transitions using TD-DFT calculations. The optimized structure of [Np^VI^O_2_(saldien)] is given in Figures S9 and S12, and selected structural parameters are summarized in Table S2. The spin-density plot of [Np^VI^O_2_(saldien)] (Figure 5a) clearly shows that the unpaired electron is predominantly located on the Np center and that the spin density value on Np is 1.18, indicating that Np has a 5f^1^ electronic configuration, the most common state for a Np^VI^O_2_^2+^ species. Using the DFT-optimized structure, the electronic absorption spectrum of [Np^VI^O_2_(saldien)] was calculated by TD-DFT calculations (black vertical lines in Figure 6). TD-DFT calculations reproduced the experimental spectral features well, but overall the transition energies were overestimated (Figure 6). Several intense bands observed at around 31000 cm^–1^ can be attributed to π–π* transitions within saldien^2–^ and ligand-to-metal charge-transfer (LMCT) from the saldien^2–^ moiety to Np (peaks (i) and (ii) in Figure 6). In addition, the TD-DFT calculations assigned the broad absorption band in the 22000–11000 cm^–1^ spectral range to LMCT from the π orbital of the saldien^2–^ moiety to the unoccupied 5f orbital of Np (peaks (iii) and (iv) in Figure 6).

Upon one-electron reduction of [Np^VI^O_2_(saldien)], the absorption band at 29000 cm^–1^ is significantly red-shifted to 27000 cm^–1^. Furthermore, a new characteristic band emerges at 10600 cm^–1^ (λ = 943 nm, ε = 550 M^–1^·cm^–1^) in [NpO_2_(saldien)]^−^ (Figure 6). This characteristic band is similar to the previously reported principal f-f transition of Np^V^O_2_^+^ from ^3^H_4g_ to ^3^Π_2g_.^54,55^ Hence, we wondered if the one-electron reduction of [Np^VI^O_2_(saldien)] would provide a Np^V^O_2_^+^ complex. In order to theoretically examine our hypothesis, we performed DFT and TD-DFT calculations on [NpO_2_(saldien)]^−^. The DFT-optimized structure of [NpO_2_(saldien)]^−^ is given in Figures S9 and S12, and selected structural parameters are summarized in Table S2. The Np≡O bond lengths, i.e., Np(1)–O(1) and Np(1)–O(2) (1.82–1.83 Å), are ca. 0.06 Å longer than those of [Np^VI^O_2_(saldien)] (Table S2). These Np≡O bond distances are similar to those of previously reported Np^V^O_2_^+^ complexes.^34,46,56^ The spin-density plot of [NpO_2_(saldien)]^−^ clearly shows that the unpaired electron is mainly distributed on the Np center (Figure 5b) and that the spin-density value on Np (2.18) indicates that Np has a 5f^2^ electronic configuration, the most common state of Np^V^O_2_^+^. The band positions and relative intensities of [Np^V^O_2_(saldien)]^−^ were theoretically examined using TD-DFT calculations (red vertical lines in Figure 6) and compared to the experimentally obtained UV-vis-NIR spectra. The intense π–π* transitions of saldien^2–^ in [Np^V^O_2_(saldien)]^−^ were calculated to be approximately 2000 cm^–1^ lower in energy than the corresponding transitions in [Np^VI^O_2_(saldien)], which is in good agreement with the experimental UV–vis-NIR observations. Consequently, [NpO_2_(saldien)]^−^ can be categorized as a Np^V^O_2_^+^ complex.

**Figure 7:**
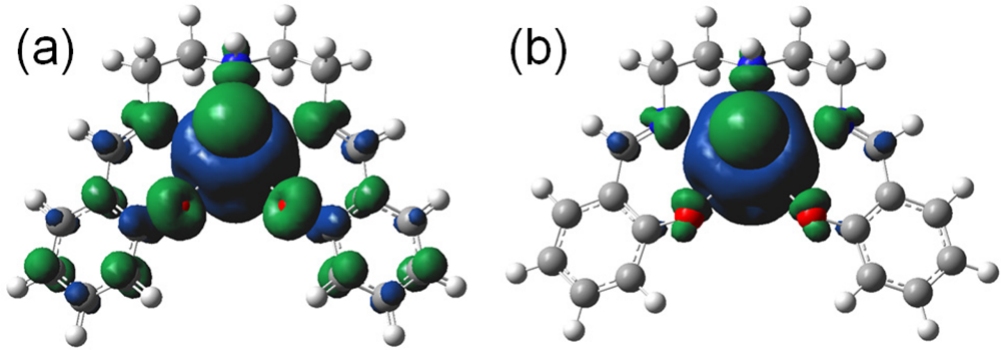
Spin-density plots of (a) [Pu^VI^O_2_(saldien)] and (b) [Pu^V^O_2_(saldien)]^−^.

**Figure 8:**
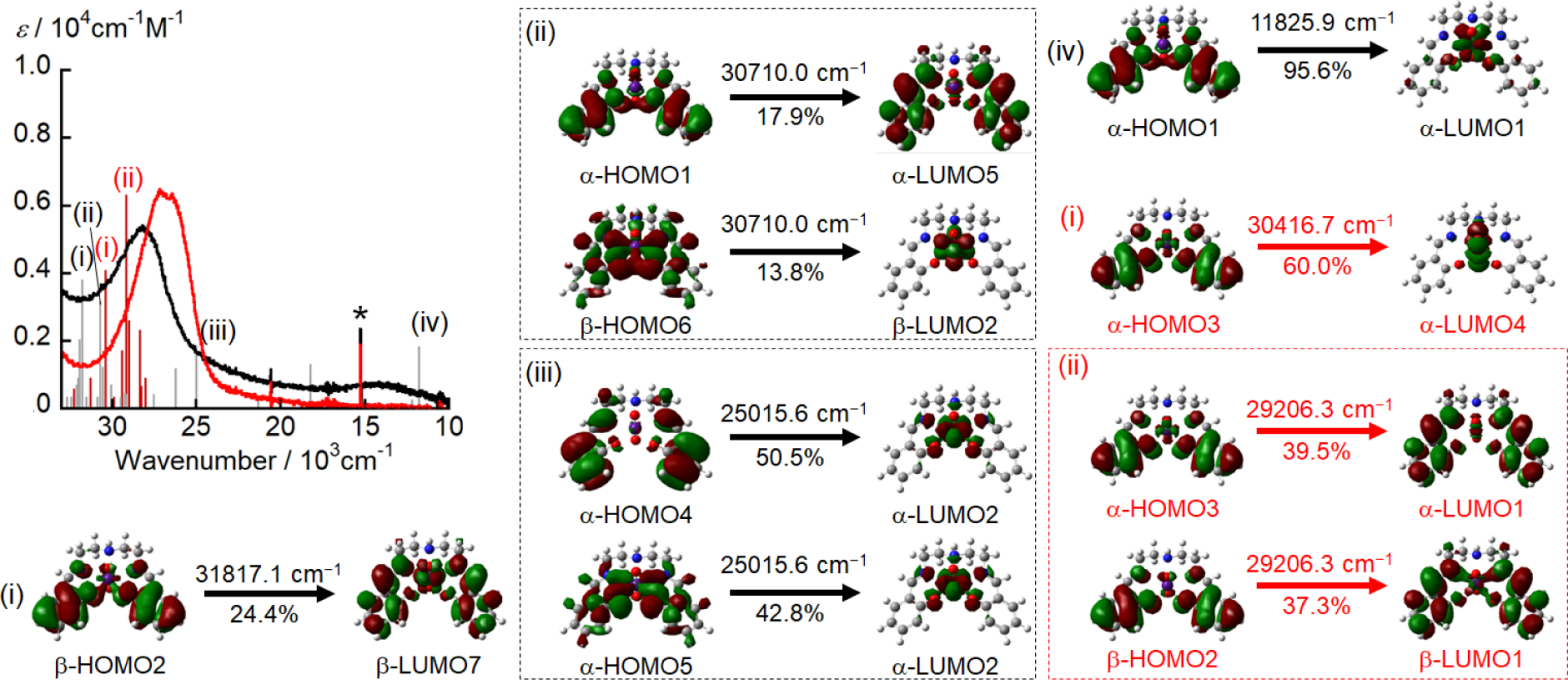
UV-vis-NIR spectra of [Pu^VI^O_2_(saldien)] (black line) and [Pu^V^O_2_(saldien)]^−^ (red line) in pyridine and the band positions and intensities predicted by TD-DFT calculations. The vertical gray and red lines correspond to the calculated transitions for [Pu^VI^O_2_(saldien)] and [Pu^V^O_2_(saldien)]^−^, respectively.

We also conducted a similar study on the redox behavior of [Pu^VI^O_2_(saldien)]. The UV-vis-NIR spectrum of [Pu^VI^O_2_(saldien)] (Figure 4b) shows an intense band at 28000 cm^–1^ (ε = 5300 M^–1^·cm^–1^) and a broad band in the 23000–10000 cm^–1^ spectral range (ε = 700 M^–1^·cm^–1^). Upon one-electron reduction of [Pu^VI^O_2_(saldien)], the absorption band at 28000 cm^–1^ was significantly red-shifted to 27000 cm^–1^ and the intensity of absorption increased. To better understand the spectral changes related to this [PuO_2_(saldien)]^−/0^ redox process, we carried out theoretical calculations for [Pu^VI^O_2_(saldien)] and [PuO_2_(saldien)]^−^ in a manner similar to those conducted for [NpO_2_(saldien)]^−/0^. The optimized structures of [Pu^VI^O_2_(saldien)] and [PuO_2_(saldien)]^−^ are given in Figures S10 and S13, and selected structural parameters are summarized in Table S3. The spin-density plot of [Pu^VI^O_2_(saldien)] (Figure 7a) clearly shows that the unpaired electron is predominantly localized on the Pu center with the spin-density value on Pu being 2.43 indicating 5f^2^ electronic configuration, the most common state for Pu^VI^O_2_^2+^. The TD-DFT calculations for [Pu^VI^O_2_(saldien)] reproduced the experimentally observed spectral features well (black vertical lines in Figure 8). Several intense bands at around 30000 cm^–1^ can be attributed to the π–π* transitions in saldien^2–^ and LMCT from saldien^2–^ to Pu (peaks (i) and (ii) in Figure 8). In addition, the broad absorption band in the 22000–10000 cm^–1^ spectral range was assigned to LMCT from the π orbital of saldien^2–^ to the unoccupied 5f orbital of Pu (peaks (iii) and (iv) in Figure 8). These spectral features are similar to those described for [Np^VI^O_2_(saldien)] above (black lines in Figure 6). [Pu^VI^O_2_(saldien)] has an Pu center with a 5f^2^ electronic configuration which is isoelectronic with [Np^V^O_2_(saldien)]^−^. However, the spectral features of these two complexes differ significantly, especially the characteristic band at 10600 cm^–1^, which is observed in [Np^V^O_2_(saldien)]^−^ but not in [Pu^VI^O_2_(saldien)].

Upon one-electron reduction of [Pu^VI^O_2_(saldien)], the absorption band at 28000 cm^–1^ was significantly red-shifted to 27000 cm^–1^. Furthermore, the broad absorption band in the 22000–10000 cm^–1^ spectral range disappeared. To understand the electronic structure of [PuO_2_(saldien)]^−^, we performed DFT and TD-DFT calculations. The DFT-optimized structure of [PuO_2_(saldien)]^−^ is given in Figures S10 and S13, and selected structural parameters are summarized in Table S3. The Pu≡O bond lengths, i.e., Pu(1)–O(1) and Pu(1)–O(2) (1.82 and 1.83 Å) are ca. 0.06 Å longer than those of the corresponding [Pu^VI^O_2_(saldien)] complex (1.76 and 1.77 Å). The elongation of the An≡O bond is frequently observed upon the reduction of An^VI^O_2_^2+^ to An^V^O_2_^+^, in which an additional electron in An^V^O_2_^+^ typically enters a nonbonding 5f orbital therefore significantly reduces the effective charge of actinide center resulting in elongating the An≡O distance.^7−9,21^ The bond lengths of the Pu center and the phenolic O atom in [PuO_2_(saldien)]^−^ (2.38 Å; Table S3) are ca. 0.13 Å longer than those of the [Pu^VI^O_2_(saldien)] complex (2.25 Å). The observed lengthening of the Pu(1)–O(3) bond suggests that the coordination bond is weakened by a decrease in the positive charge of the Pu atom through the reduction of Pu^VI^O_2_^2+^ to Pu^V^O_2_^+^. The spin-density plot of [PuO_2_(saldien)]^−^ clearly shows that the unpaired electron spin is mainly distributed on the Pu center (Figure 7b). The spin-density value of Pu (3.31) indicates that Pu has a 5f^3^ electronic configuration, the most common state for Pu^V^O_2_^+^.

The band positions and relative intensities of [Pu^V^O_2_(saldien)]^−^ were theoretically examined using TD-DFT calculations (red vertical lines in Figure 8) and compared to the experimentally obtained UV-vis-NIR spectra. The intense π–π* transitions of the saldien^2–^ ligand and the LMCT bands in [Pu^V^O_2_(saldien)]^−^ were calculated to be approximately 1500 cm^–1^ lower in energy than the corresponding transitions in [Pu^VI^O_2_(saldien)], which is in good agreement with the experimentally observed UV-vis-NIR features. A similarly significant red-shift of the intense absorption bands was also observed in the previously discussed reduction of [Np^VI^O_2_(saldien)] to [Np^V^O_2_(saldien)]^−^. Consequently, we conclude that the one-electron reduction of [Pu^VI^O_2_(saldien)] results in the formation of the Pu^V^O_2_^+^ complex [Pu^V^O_2_(saldien)]^−^.

As described in the introduction, the oxygen atoms of the An^V^O_2_^+^ species prefer to interact via CCIs as equatorial ligands with the An centers of other An^V^O_2_^+^ species.^28−30,33,34^ Indeed, all Np^V^O_2_^+^ complexes with Schiff-base ligands reported to date form oligomeric or polymeric species.^33,34^ The spectroelectrochemical measurements of the [Np^V/VI^O_2_(saldien)]^−/0^ and [Pu^V/VI^O_2_(saldien)]^−/0^ couples revealed the presence of clear isosbestic points, indicating that the reduction process from An^VI^O_2_^2+^ to An^V^O_2_^+^ takes place without any oligomerization or polymerization caused by CCIs. Furthermore, reversibility of cyclic voltammograms in both systems (Figure 3) also corroborates absence of any successive reactions such as polynucleation of An^V^O_2_^+^ species through their CCIs. The [Np^V^O_2_(saldien)]^−^ and [Pu^V^O_2_(saldien)]^−^ complexes described here thus represent the first examples of spectroscopically characterized mononuclear Np^V^O_2_^+^ and Pu^V^O_2_^+^ complexes with Schiff-base ligands.

### Redox Trends in the [An^VI^O_2_(saldien)] Series (An = U, Np, Pu)

In this section, we discuss the trends observed in [An^V/VI^O_2_(saldien)]^−/0^ redox processes. The CV measurements demonstrated that the *E*°′ value of the [U^V/VI^O_2_(saldien)]^−/0^ couple (−1.667 V vs Fc^0/+^) is significantly more negative than those of the [Np^V/VI^O_2_(saldien)]^−/0^ (−0.650 V) and [Pu^V/VI^O_2_(saline)]^−/0^ (−0.698 V) couples (Figure S14). To understand this trend in *E*°′ values, we carried out a theoretical investigation of the [An^V/VI^O_2_(saldien)]^−/0^ redox processes. Initially, the redox potentials of the [An^V/VI^O_2_(saldien)]^−/0^ couples were estimated using quantum chemical calculations and subsequently compared to the experimentally determined redox potentials (*E*°′). The theoretical estimation of the redox potentials was conducted with respect to the reference system, ferrocenium/ferrocene (Fc^0/+^), through the application of the following reaction and equations.
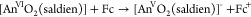

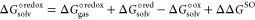
2
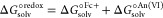
3
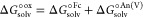
4
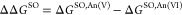
5
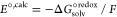
6where Δ and Δ are the standard Gibbs free energy of the redox half reaction in solution and the gas phase, respectively, while Δ and Δ are the solvation free energies of the reduced forms and oxidized forms, respectively. Δ*G*^SO,An(V)^ and Δ*G*^SO,An(VI)^ refer to the spin–orbit effect of An^V^O_2_^+^ and An^VI^O_2_^2+^ complexes relative to the spin-free CASSCF ground state. For the spin–orbit effect, the previously reported values for the [An^V/VI^O_2_(H_2_O)_5_]^+/2+^ complexes were used.^57^*E*°^,calc^ is the theoretically estimated standard redox potential. The estimated *E*°^,calc^ values of the [U^V/VI^O_2_(saldien)]^−/0^ (−1.779 V), [Np^V/VI^O_2_(saldien)]^−/0^ (−0.728 V), and [Pu^V/VI^O_2_(saldien)]^−/0^ couples (−0.887 V) are 0.1–0.2 V more negative than the corresponding experimental values (*E*°′ = −1.580 V, – 0.650 V and −0.698 V, respectively). Although the *E*°^,calc^ values slightly deviate from the actual experimental values (*E*°′), the trend in *E*°^,calc^ of [An^V/VI^O_2_(saldien)]^−/0^ series reproduces the experimentally observed trend (Figure S14).

Here, it is important to highlight that a key distinction between the [U^VI^O_2_(saldien)] complex and the other [An^VI^O_2_(saldien)] complexes is the presence or absence of 5f electrons. All the 5f orbitals of U in [U^VI^O_2_(saldien)] are empty, while the 5f orbitals of Np and Pu in the other [An^VI^O_2_(saldien)] complexes are partially occupied. When [U^VI^O_2_(saldien)] is reduced to [U^V^O_2_(saldien)]^−^, the electronic configuration changes from a closed-shell 5f^0^ to an open-shell 5f^1^. In the case of Np and Pu, the + VI oxidation state already has an open-shell electronic configuration. Furthermore, the + V oxidation state with 5f electron occupations greater than 2 is strongly multiconfigurational. It appears to be easier to add an additional electron to the [An^VI^O_2_(saldien)] complexes (An = Np and Pu) with an open-shell electronic configuration and multiconfigurational in nature rather than to add an electron to the closed-shell and stable [U^VI^O_2_(saldien)] compound. The experimental and theoretical results indicate that [Pu^VI^O_2_(saldien)] is more readily reduced to the + V oxidation state than [Np^VI^O_2_(saldien)]. It is difficult to elucidate the difference in redox potentials between the two systems, as multitude factors, including electronic energy, spin–orbit coupling, entropy contribution, and solvation energy, exert influence on the redox potential. The primary distinction between the redox potentials between the [Np^V/VI^O_2_(saldien)]^−/0^ and [Pu^V/VI^O_2_(saldien)]^−/0^ couples can be attributed to the variation in spin–orbit coupling.^57^ Regardless, in order to accurately and systematically understand the redox behavior of An^VI^O_2_^2+^ complexes, it is important to carefully discuss a combination of experimental and computational results.

## Conclusion

In this study, we aimed to gain a comprehensive and systematic understanding of An^VI^O_2_^2+^ coordination chemistry and redox behavior. For that purpose, [An^VI^O_2_(saldien)] complexes (An = Np, Pu) were synthesized and characterized and their redox behavior was investigated both experimentally and theoretically. The molecular structures of these [An^VI^O_2_(saldien)] complexes (An = U, Np, Pu) are comparable. As expected, the saldien^2–^ ligand saturates the equatorial plane of the An^VI^O_2_^2+^ species to fully preclude any further coordination to other An centers via cation–cation interactions (CCIs). In contrast, the redox potentials of these complexes strongly depend on the nature of the An center. In pyridine, [Np^VI^O_2_(saldien)] and [Pu^VI^O_2_(saldien)] both exhibit one redox couple at −0.650 V and −0.698 V vs Fc^0/+^, respectively. These *E*°′ values are significantly more positive than the *E*°′ value of [U^V/VI^O_2_(saldien)]^−/0^ (−1.667 V). By combining spectroelectrochemical measurements with density-functional-theory (DFT) and time-dependent density-functional-theory (TD-DFT) calculations, the redox equilibria observed in [Np^VI^O_2_(saldien)] and [Pu^VI^O_2_(saldien)] were assigned to the [Np^V/VI^O_2_(saldien)]^−/0^ and [Pu^V/VI^O_2_(saldien)]^−/0^ couples. The difference in *E*°′ between the [An^V/VI^O_2_(saldien)]^−/0^ couples can be explained in terms of the difference in character of the electronic configuration of the + VI oxidation state. The reduction of the closed-shell [U^VI^O_2_(saldien)] complex is more difficult than that of the other [An^VI^O_2_(saldien)] complexes (An = Np and Pu) which have open-shell electronic configurations and multiconfigurational in nature. Furthermore, we successfully reproduced the redox trend of a series of [An^V/VI^O_2_(saldien)]^−/0^ experimentally observed here, through computational approaches using DFT.

In this study, we have for the first time experimentally demonstrated the systematic trends in the structural and redox chemistry of An^VI^O_2_^2+^ complexes with Schiff-base ligands. The present findings provide valuable information for controlling the oxidation states of An elements in the environment and in nuclear-fuel cycles. A deeper and systematic understanding of An^VI^O_2_^2+^ complexes will open up the path to applications in spintronics that utilize the anisotropic 5f electron spins.

## Supporting Information Available

IR spectra, electrochemical data, optimized structures, and structural parameters of the [An^VI^O_2_(saldien)] and [An^V^O_2_(saldien)]^−^ complexes. Crystallographic data reported in this manuscript have been deposited at the Cambridge Crystallographic Data Centre as supplementary publication no. CCDC 2383153 for [Np^VI^O_2_(saldien)] and 2383152 for [Pu^VI^O_2_(saldien)] (PDF)
