# Supplementary material for: A Series of AnVIO22+ Complexes (An = U, Np, Pu) with N3O2-Donating Schiff-Base Ligands: Systematic Trends in the Molecular Structures and Redox Behavior
Source: Inorg Chem. 2025 Jan 3;64(3):1313–22. doi: 10.1021/acs.inorgchem.4c04185 (PMC11776050; doi:10.1021/acs.inorgchem.4c04185)
Supplement: Supplementary file 1 — ic4c04185_si_001.pdf [file ic4c04185_si_001.pdf]

Supporting Information

A Series of $An^{VI}O_2^{2+}$ Complexes (An = U, Np, Pu) with N_3O_2-Donating Schiff-Base Ligands: Systematic Trends in the Molecular Structures and Redox Behavior

Tomoyuki Takeyama,*[a],[b] Satoru Tsushima,[b],[c] Robert Gericke,[c] Tamara M. Duckworth,[c] Peter Kaden,[c] Juliane März,[c] and Koichiro Takao*[b]

[a] Department of Applied Chemistry, Sanyo-Onoda City University, 1-1-1, Daigakudori, Sanyo-Onoda, Yamaguchi 756-0884, Japan

E-mail: takeyama.t@rs.socu.ac.jp

[b] Laboratory for Zero-Carbon Energy, Institute of Integrated Research, Institute of Science Tokyo, 2-12-1 N1-32, O-okayama, Meguro-ku, 152-8550 Tokyo, Japan

E-mail: ktakao@zc.iir.isct.ac.jp.

[c] Institute of Resource Ecology, Helmholtz-Zentrum Dresden-Rossendorf (HZDR) Bautzner Landstraße 400, 01328 Dresden, Germany

Table S1. Selected bond lengths (Å) of DFT-optimized structures of [$U^{VI}O_2$(saldien)] and [$U^{V}O_2$(saldien)]$^{-}$.

[$U^{VI}O_2$(saldien)]		[$U^{V}O_2$(saldien)]$^{-}$	
Vacuum space			
U(1)−O(1)	1.791	U(1)−O(1)	1.843
U(1)−O(2)	1.789	U(1)−O(2)	1.841
U(1)−O(3)	2.245	U(1)−O(3)	2.400
U(1)−N(1)	2.658	U(1)−N(1)	2.734
U(1)−N(2)	2.670	U(1)−N(2)	2.702
C(1)−O(3)	1.301	C(1)−O(3)	1.275
In Pyridine (CPCM)			
U(1)−O(1)	1.798	U(1)−O(1)	1.856
U(1)−O(2)	1.795	U(1)−O(2)	1.853
U(1)−O(3)	2.254	U(1)−O(3)	2.391
U(1)−N(1)	2.633	U(1)−N(1)	2.687
U(1)−N(2)	2.643	U(1)−N(2)	2.674
C(1)−O(3)	1.311	C(1)−O(3)	1.291

Table S2. Selected bond lengths (Å) of DFT-optimized structures of [$Np^{VI}O_2$(saldien)] and [$Np^{V}O_2$(saldien)]$^{-}$.

[$Np^{VI}O_2$(saldien)]		[$Np^{V}O_2$(saldien)]$^{-}$	
Vacuum space			
Np(1)−O(1)	1.770	Np(1)−O(1)	1.824
Np(1)−O(2)	1.767	Np(1)−O(2)	1.820
Np(1)−O(3)	2.231	Np(1)−O(3)	2.406
Np(1)−N(1)	2.641	Np(1)−N(1)	2.727
Np(1)−N(2)	2.673	Np(1)−N(2)	2.700
C(1)−O(3)	1.301	C(1)−O(3)	1.272
In Pyridine (CPCM)			
Np(1)−O(1)	1.771	Np(1)−O(1)	1.825
Np(1)−O(2)	1.767	Np(1)−O(2)	1.822
Np(1)−O(3)	2.261	Np(1)−O(3)	2.383
Np(1)−N(1)	2.601	Np(1)−N(1)	2.672
Np(1)−N(2)	2.642	Np(1)−N(2)	2.664
C(1)−O(3)	1.309	C(1)−O(3)	1.290

Table S3. Selected bond lengths (Å) of DFT-optimized structures of [$Pu^{VI}O_2$(saldien)] and [$Pu^{V}O_2$(saldien)]$^{-}$.

[$Pu^{VI}O_2$(saldien)]		[$Pu^{V}O_2$(saldien)]$^{-}$	
Vacuum space			
Pu(1)−O(1)	1.753	Pu(1)−O(1)	1.810
Pu(1)−O(2)	1.751	Pu(1)−O(2)	1.807
Pu(1)−O(3)	2.239	Pu(1)−O(3)	2.392
Pu(1)−N(1)	2.604	Pu(1)−N(1)	2.713
Pu(1)−N(2)	2.679	Pu(1)−N(2)	2.687
C(1)−O(3)	1.300	C(1)−O(3)	1.272
In Pyridine (CPCM)			
Pu(1)−O(1)	1.768	Pu(1)−O(1)	1.827
Pu(1)−O(2)	1.761	Pu(1)−O(2)	1.824
Pu(1)−O(3)	2.247	Pu(1)−O(3)	2.382
Pu(1)−N(1)	2.604	Pu(1)−N(1)	2.666
Pu(1)−N(2)	2.633	Pu(1)−N(2)	2.653
C(1)−O(3)	1.310	C(1)−O(3)	1.291

Table S4. Electrochemical data of [$U^{VI}O_2$(saldien)] in pyridine containing 0.1 M *tetra-n*-butylammonium perchlorate at 295 K.

Scan rate	E_{pc} / V[a]	E_{pa} / V[a]	$E°'$ / V[a]	ΔE_p / mV
50 $mV·s^{-1}$	−1.617	−1.720	−1.669	103
100 $mV·s^{-1}$	−1.610	−1.723	−1.667	113
200 $mV·s^{-1}$	−1.600	−1.733	−1.667	133
500 $mV·s^{-1}$	−1.587	−1.757	−1.672	170

[a] vs. $Fc^{0/+}$

Table S5. Electrochemical data of [$Np^{VI}O_2$(saldien)] in pyridine containing 0.1 M *tetra-n*-butylammonium perchlorate at 295 K.

Scan rate	E_{pc} / V[a]	E_{pa} / V[a]	$E°'$ / V[a]	ΔE_p / mV
50 $mV·s^{-1}$	−0.610	−0.690	−0.650	80
100 $mV·s^{-1}$	−0.605	−0.695	−0.650	90
200 $mV·s^{-1}$	−0.600	−0.700	−0.650	100
400 $mV·s^{-1}$	−0.595	−0.710	−0.653	115
600 $mV·s^{-1}$	−0.585	−0.715	−0.650	130
800 $mV·s^{-1}$	−0.580	−0.720	−0.650	140
1000 $mV·s^{-1}$	−0.575	−0.725	−0.650	150

[a] vs. $Fc^{0/+}$

Table S6. Electrochemical data of [$Pu^{VI}O_2$(saldien)] in pyridine containing 0.1 M *tetra-n*-butylammonium perchlorate at 295 K.

Scan rate	E_{pc} / V[a]	E_{pa} / V[a]	$E°'$ / V[a]	ΔE_p / mV
50 $mV·s^{-1}$	−0.658	−0.751	−0.704	93
75 $mV·s^{-1}$	−0.652	−0.751	−0.702	99
100 $mV·s^{-1}$	−0.650	−0.745	−0.698	95
120 $mV·s^{-1}$	−0.652	−0.751	−0.701	99

[a] vs. $Fc^{0/+}$

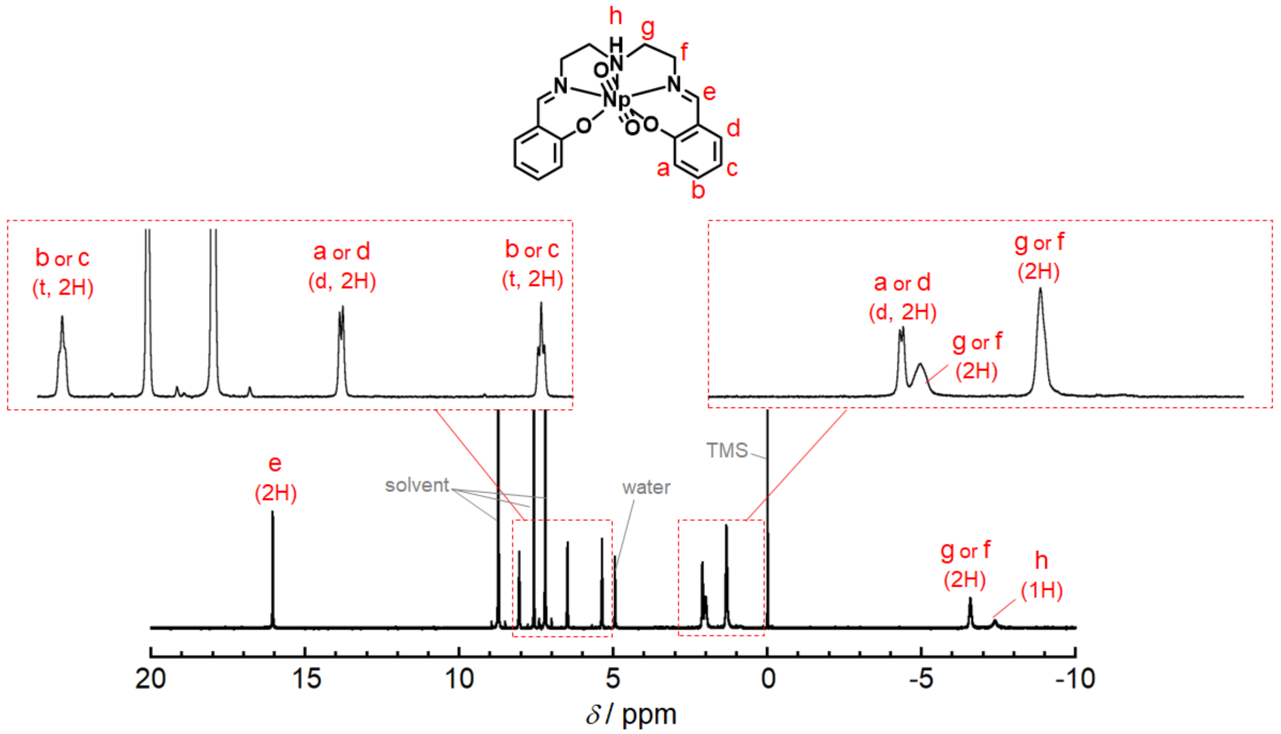

Figure S1. ^{1}H NMR spectrum of $[Np^{VI}O_2(saldien)]$ in pyridine-d_5.

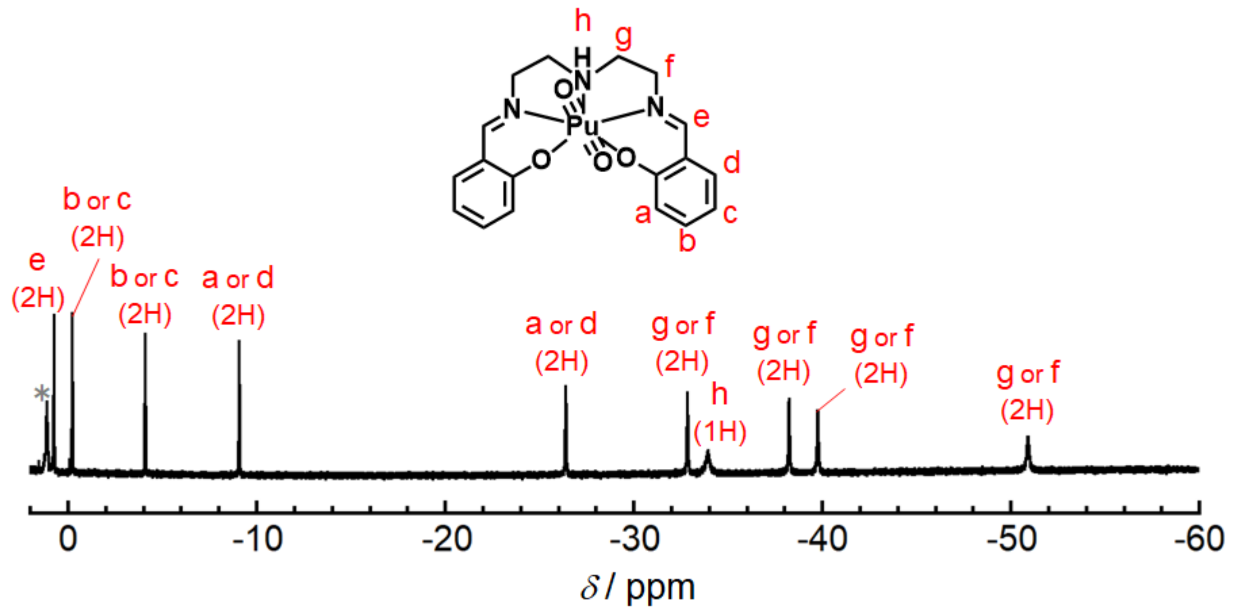

Figure S2. ^{1}H NMR spectrum of $[Pu^{VI}O_2(saldien)]$ in pyridine-d_5. Gray asterisk is attributed to residual crystalline solvent (diethyl ether).

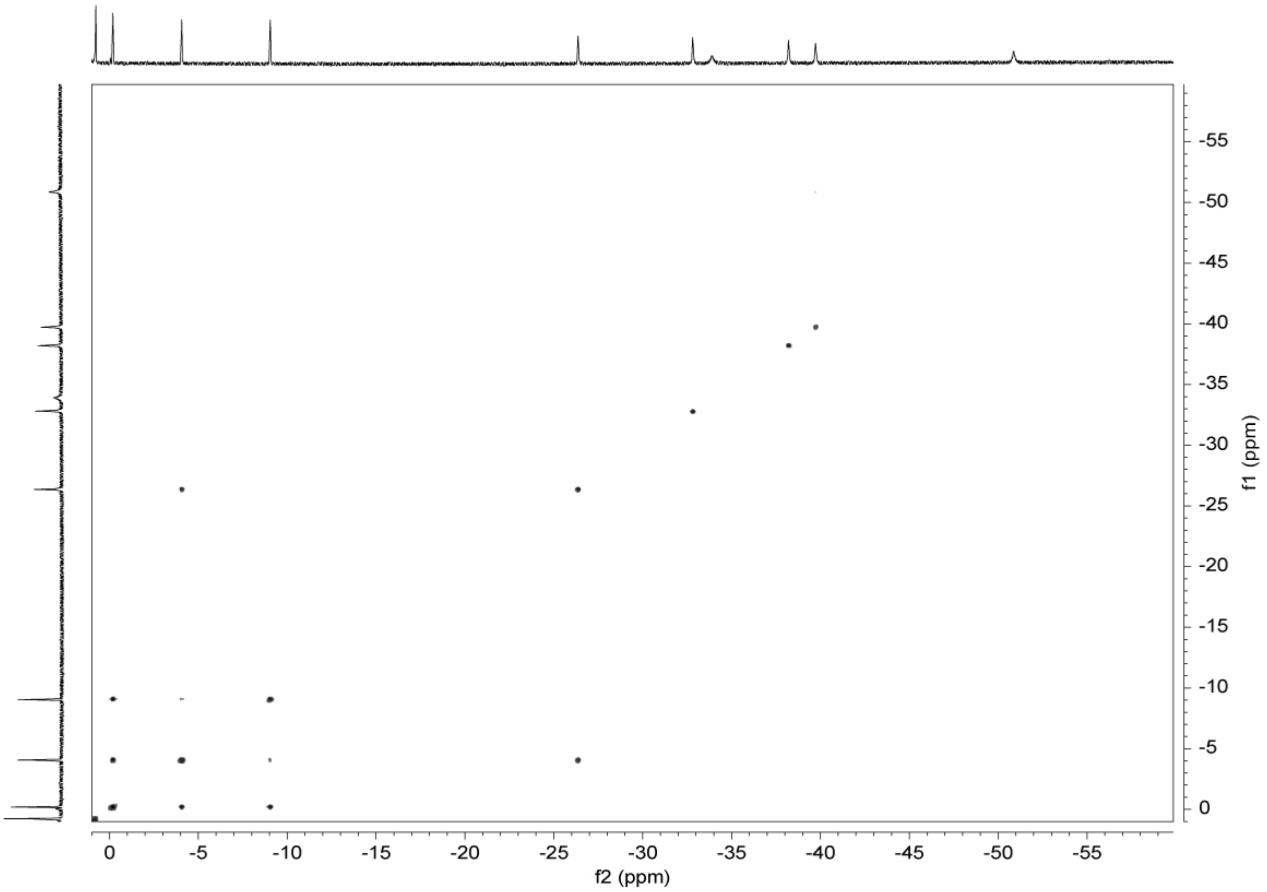

Figure S3. ^{1}H-^{1}H COSY NMR spectrum of [$Pu^{VI}O_2$(saldien)] in pyridine-d_5.

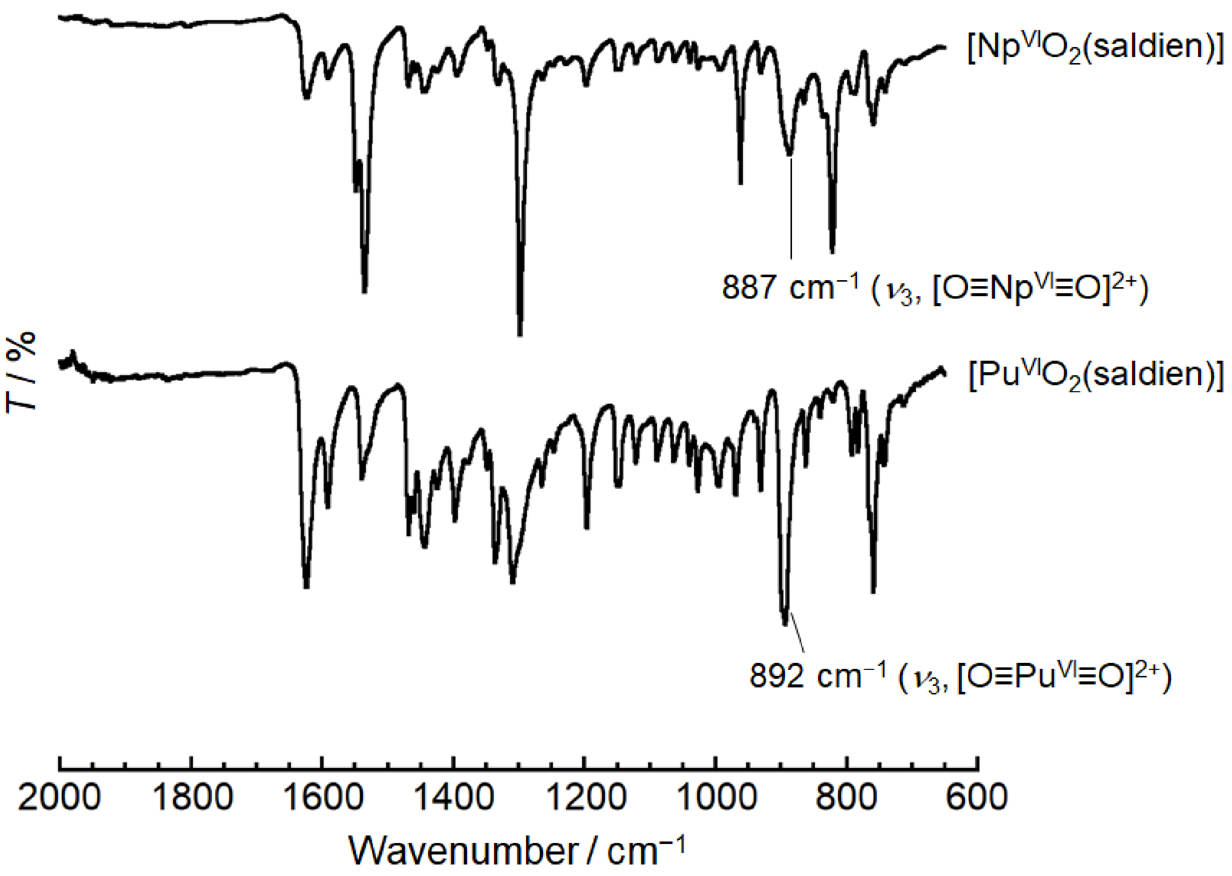

Figure S4. IR spectra of $[Np^{VI}O_2(saldien)]$ and $[Pu^{VI}O_2(saldien)]$ in solid state.

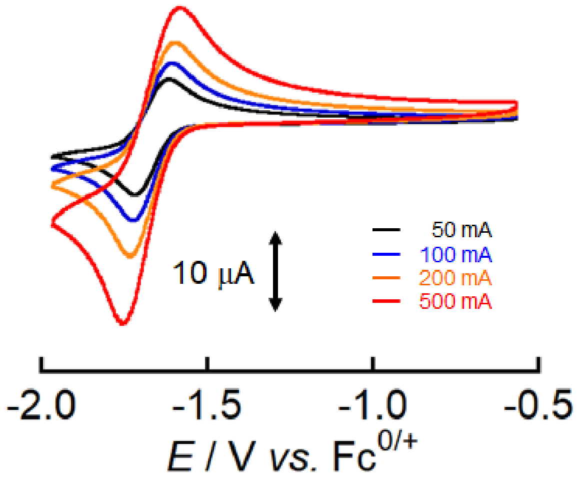

Figure S5. Cyclic voltammograms for the redox couples of $[UO_2(saldien)]^{-/0}$ in pyridine at 295 K. Concentration of the complex was adjusted to 1 mM. Tetra-*n*-butylammonium perchlorate (0.1 M) was used as a supporting electrolyte. Potentials in the figures show the relative values to that of the $Fc^{0/+}$ redox couple. Scan rates are 50 $mV{\cdot}s^{-1}$ (black), 100 $mV{\cdot}s^{-1}$ (blue), 200 $mV{\cdot}s^{-1}$ (orange) and 500 $mV{\cdot}s^{-1}$ (red).

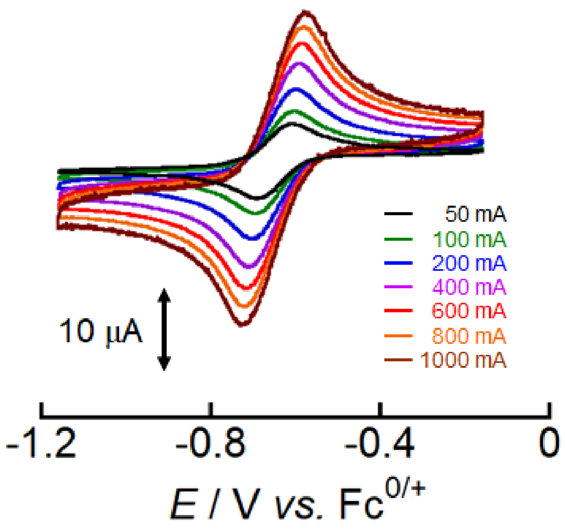

Figure S6. Cyclic voltammograms for the redox couples of $[NpO_2(saldien)]^{-/0}$ in pyridine at 295 K. Concentration of the complex was adjusted to 1 mM. Tetra-*n*-butylammonium perchlorate (0.1 M) was used as a supporting electrolyte. Potentials in the figures show the relative values to that of the $Fc^{0/+}$ redox couple. Scan rates are 50 $mV{\cdot}s^{-1}$ (black), 100 $mV{\cdot}s^{-1}$ (green), 200 $mV{\cdot}s^{-1}$ (blue), 400 $mV{\cdot}s^{-1}$ (pink), 600 $mV{\cdot}s^{-1}$ (red), 800 $mV{\cdot}s^{-1}$ (orange) and 1000 $mV{\cdot}s^{-1}$ (brown).

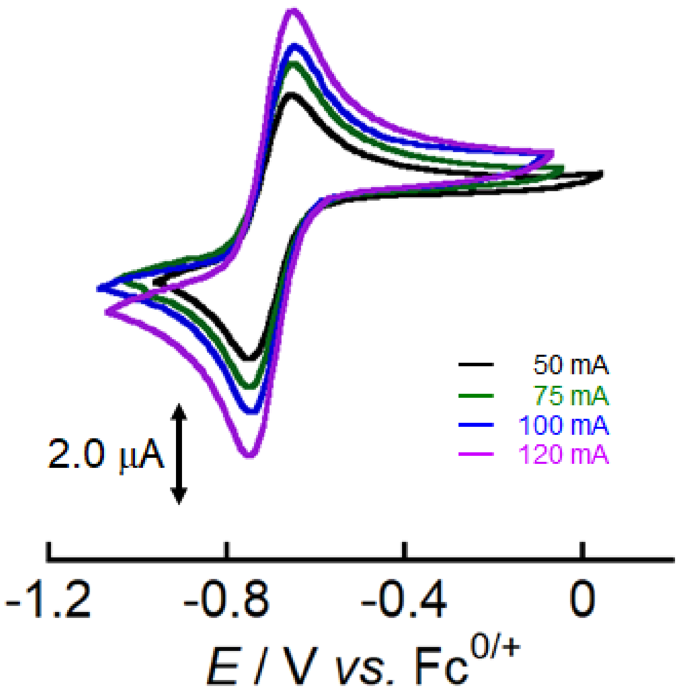

Figure S7. Cyclic voltammograms for the redox couples of $[PuO_2(saldien)]^{-/0}$ in pyridine at 295 K. Concentration of the complex was adjusted to 0.8 mM. Tetra-*n*-butylammonium perchlorate (0.1 M) was used as a supporting electrolyte. Potentials in the figures show the relative values to that of the $Fc^{0/+}$ redox couple. Scan rates are 50 mV·s^{-1} (black), 75 mV·s^{-1} (green), 100 mV·s^{-1} (blue) and 120 mV·s^{-1} (pink).

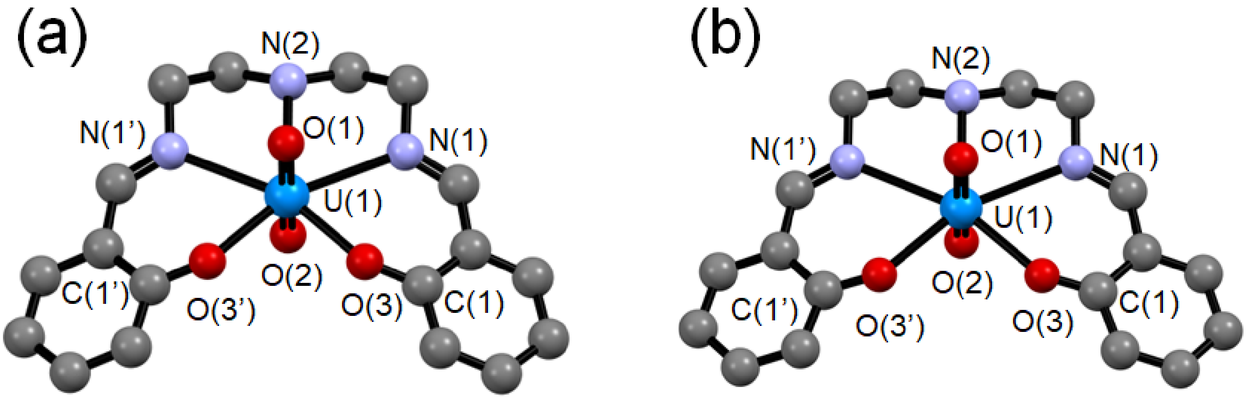

Figure S8. DFT-optimized structures in vacuum of $[U^{VI}O_2(saldien)]$ (a) and $[U^{V}O_2(saldien)]^-$ (b).

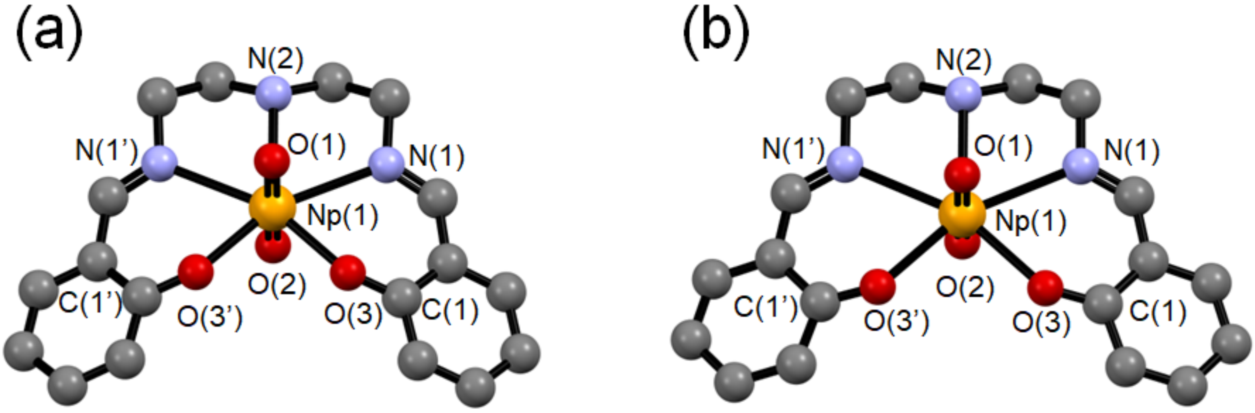

Figure S9. DFT-optimized structures in vacuum of $[Np^{VI}O_2(saldien)]$ (a) and $[Np^{V}O_2(saldien)]^-$ (b).

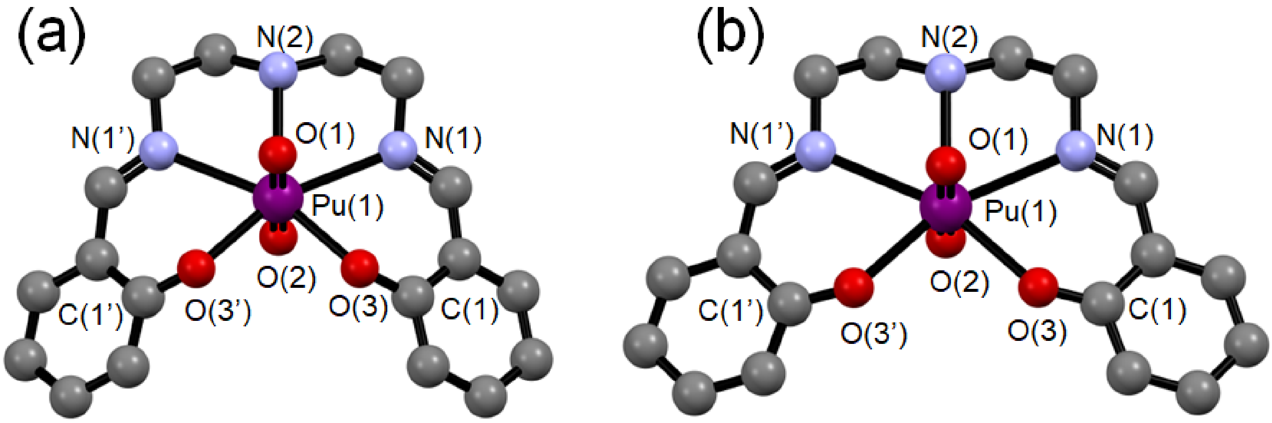

Figure S10. DFT-optimized structures in vacuum of $[Pu^{VI}O_2(saldien)]$ (a) and $[Pu^{V}O_2(saldien)]^-$ (b).

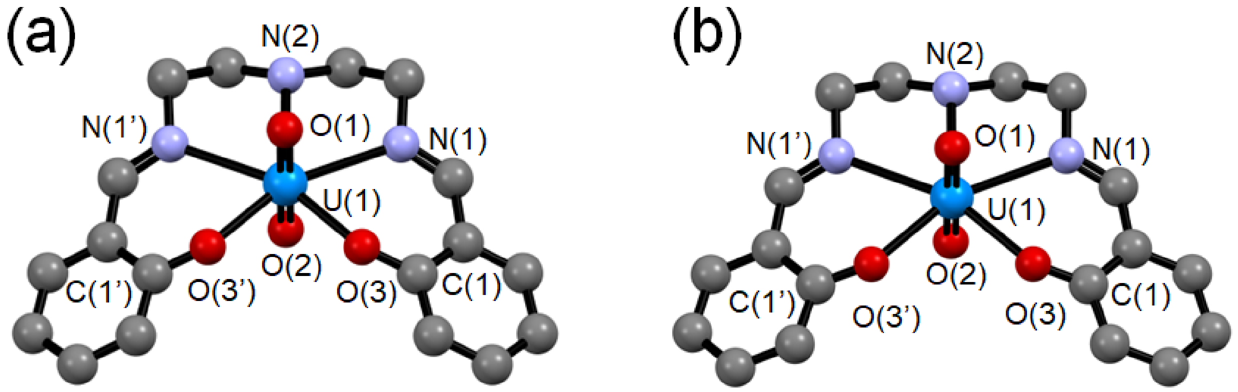

Figure S11. DFT-optimized structures in pyridine of [$U^{VI}O_2$(saldien)] (a) and [$U^{V}O_2$(saldien)]$^-$ (b).

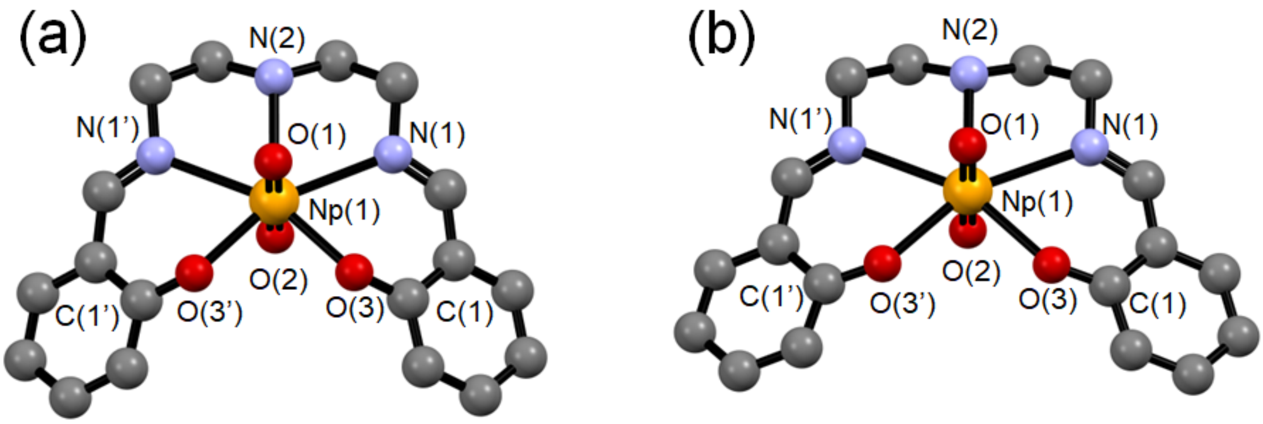

Figure S12. DFT-optimized structures in pyridine of [$Np^{VI}O_2$(saldien)] (a) and [$Np^{V}O_2$(saldien)]$^-$ (b).

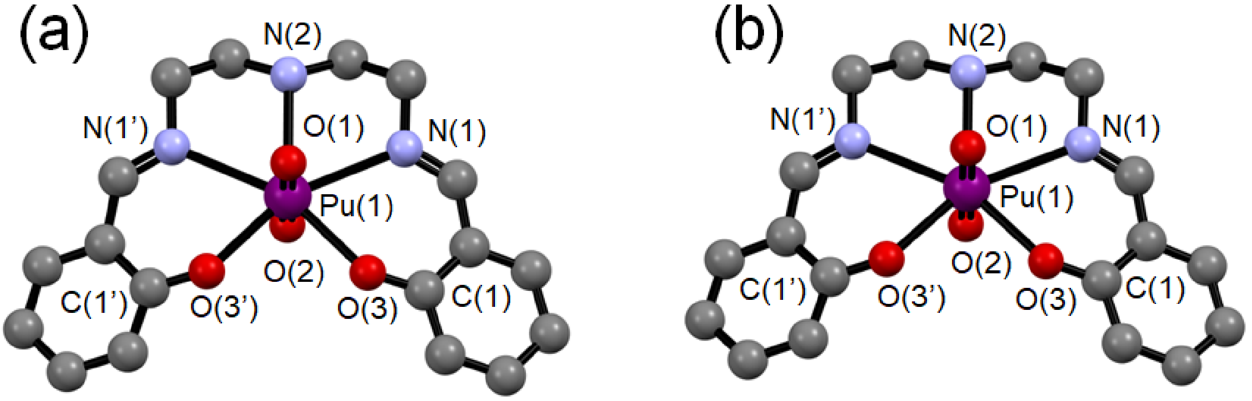

Figure S13. DFT-optimized structures in pyridine of [$Pu^{VI}O_2$(saldien)] (a) and [$Pu^{V}O_2$(saldien)]$^-$ (b).

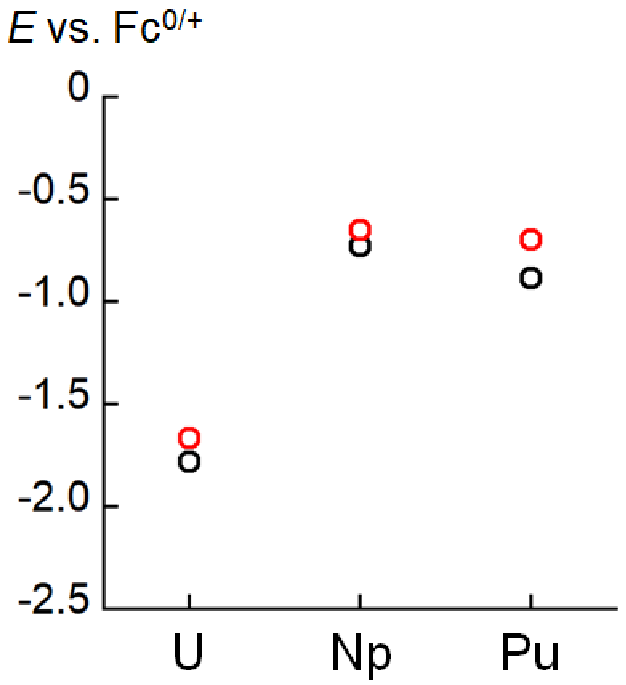

Figure S14. (a) The redox potentials the $[An^{V/VI}O_2(saldien)]^{-/0}$ couples estimated via DFT calculations ($E^{\circ,calc}$, black circles) and CV measurements ($E^{\circ\prime}$, red circles).
